# Additional Diagnostic Value of Unenhanced Computed Tomography plus Diffusion-Weighted Imaging Combined with Routine Magnetic Resonance Imaging Findings of Early-Stage Gliblastoma

**DOI:** 10.1155/2020/1672736

**Published:** 2020-02-18

**Authors:** Hexiang Wang, Zhenyou Liu, Yong Zhang, Feng Hou, Weiwei Fu, Jizheng Lin, Yingchao Liu, Xuejun Liu

**Affiliations:** ^1^Department of Radiology, The Affiliated Hospital of Qingdao University, Qingdao, Shandong, China; ^2^Department of Radiology, Qingdao Eighth People's Hospital, Qingdao, Shandong, China; ^3^Department of Radiology, The Xixiu District People's Hospital, Anshun, Guizhou, China; ^4^Department of Pathology, The Affiliated Hospital of Qingdao University, Qingdao, Shandong, China; ^5^Department of Neurosurgery, Shandong Provincial Hospital Jinan, Jinan, Shandong, China

## Abstract

**Purpose:**

This study was performed to determine whether diffusion-weighted imaging (DWI) plus unenhanced computed tomography (CT) of the brain increases the diagnostic value of routine magnetic resonance (MR) imaging findings of early-stage glioblastoma.

**Methods:**

Postcontrast MR images of eight unenhanced lesions that had been pathologically diagnosed as glioblastoma were retrospectively examined. The location, margin, signal intensity, and attenuation on MR imaging and CT were assessed.

**Results:**

On MR imaging, all lesions were ill-defined, small, and isointense to hypointense on T1-weighted images and hyperintense on T2-weighted images. Four patients had perilesional edema. In seven patients, DWI showed an inhomogeneous hyperintense lesion (*n* = 1) or isointense lesion with a hyperintense region (*n* = 1) or isointense lesion with a hyperintense region (*n* = 1) or isointense lesion with a hyperintense region (*n* = 1) or isointense lesion with a hyperintense region (*n* = 1) or isointense lesion with a hyperintense region (*n* = 1) or isointense lesion with a hyperintense region (*n* = 1) or isointense lesion with a hyperintense region (*n* = 1) or isointense lesion with a hyperintense region (*n* = 1) or isointense lesion with a hyperintense region (

**Conclusions:**

MR imaging was the most sensitive imaging method for depicting early-stage glioblastoma. The CT finding of a hyperattenuated or isoattenuated region combined with the DWI finding of the same region containing an inhomogeneous hyperintense lesion or isointense lesion with a hyperintense region may be a specific diagnostic sign for early-stage glioblastoma. DWI plus unenhanced CT added diagnostic value to the routine MR imaging findings of early-stage glioblastoma.

## 1. Background

Glioblastoma is the most common primary intracranial neoplasm in adults, accounting for about 15% to 20% of all intracranial tumors [1]. Glioblastoma is commonly diagnosed based on the typical magnetic resonance (MR) imaging appearance of significant enhanced with heterogeneous-ring mass lesions with obvious peritumoral brain edema, hemorrhage, or necrosis [2]. However, a subgroup of glioblastoma is found at an earlier stage before central necrosis and lesion enhancement occur [3], and about 4% of glioblastoma masses display no enhancement at the first appearance. These lesions can be misdiagnosed as nonneoplastic cerebral lesions such as infarction, encephalitis, or degenerative or demyelinating disease [2, 4–7].

Early diagnosis of glioblastoma should provide a better prognosis because of the added potential of safe excision; however, delayed diagnosis may result in a poor clinical outcome [7]. The correct diagnosis of early-stage glioblastoma is important; however, when a mass manifests as a nonneoplastic cerebral lesion, the correct diagnosis is usually not made before progression to typical glioblastoma. Thus, challenges are associated with the use of routine MR imaging for diagnosis of early glioblastoma.

Several studies of nonneoplastic cerebral lesions such as infarction, encephalitis, and demyelinating or degenerative diseases have shown that unenhanced computed tomography (CT) reveals either mass hypoattenuation or no positive signs [8–12]. To our knowledge, however, the capability of CT to help achieve the correct diagnosis of early-stage glioblastoma has not been emphasized in previous reports. Additionally, past studies have shown that diffusion-weighted imaging (DWI) is useful for diagnosis of malignant glioma [13]. Therefore, the purpose of our study was to determine whether unenhanced CT plus DWI of the brain has added value in the diagnosis of early-stage glioblastoma.

## 2. Methods

### 2.1. Patients

This retrospective study was approved by our hospital ethics committee. In total, 225 patients with a pathologically confirmed final diagnosis of glioblastoma underwent imaging examinations from July 2003 to January 2018. Among these 225 patients, 8 with a diagnosis of glioblastoma and no enhancement on postcontrast MR imaging were reviewed in this study. The patients comprised three men and five women ranging in age from 36 to 71 years (mean, 55.3 years). Our team analyzed these patients' MR images and unenhanced CT scans in the present study.

All eight patients underwent plain MR imaging, contrast-enhanced T1WI, and unenhanced CT. The MR imaging examinations were performed with either a 1.5-T scanner (*n* = 3) (Signa Advantage Horizon; GE Medical Systems, Milwaukee, WI, USA) or a 3.0-T MR scanner (*n* = 5) (Signa HDx; GE Medical Systems). The scanning parameters for T1WI were a repetition time (TR) of 500 to 600 ms and echo time (TE) of 15 to 30 ms; those for fat-suppressed fast spin echo T2WI were a TR of 3650 to 5100 ms and TE of 85 to 120 ms; and those of contrast-enhanced MR imaging using a spin echo T1WI sequence were a TR of 500 to 650 ms and TE of 10 to 15 ms with injection of 0.2 ml/kg gadolinium dimeglumine. The slice thickness was 3 to 4 mm, the slice spacing was 1 mm, the field of view was 200 to 400 mm, and the matrix was 208 to 512 × 208 to 512. Seven patients underwent DWI (*b* = 0 and 1000 s/mm^2^). CT was performed based on the standard CT protocol for the head (field of view of 200–400 mm, matrix of 512 × 512, and slice thickness of 5–10 mm).

### 2.2. Imaging Analysis

Two neuroradiologists who had >9 years of professional experience and were blinded to the clinical data independently reviewed the imaging findings. The reviewers recorded the tumor location, lesion number (solitary or multiple), margin, signal intensity, and density. The signal intensity/density was classified as hypointense/hypodense, isointense/isodense, or hyperintense/hyperdense compared with the adjacent brain parenchyma on the MR images or unenhanced CT images, respectively. On contrast-enhanced MR imaging, the degree of enhancement was recorded as none, moderate, or marked. Other associated findings such as perilesional edema were also recorded. The imaging appearance was independently compared with the pathologic findings.

## 3. Results

For five patients in our study, the initial MR imaging examination was performed mainly for evaluation of seizures. General symptoms included mild headache and vertigo in three patients and mild hemiparesis in one. One asymptomatic patient was diagnosed incidentally.

**Figure 1:**
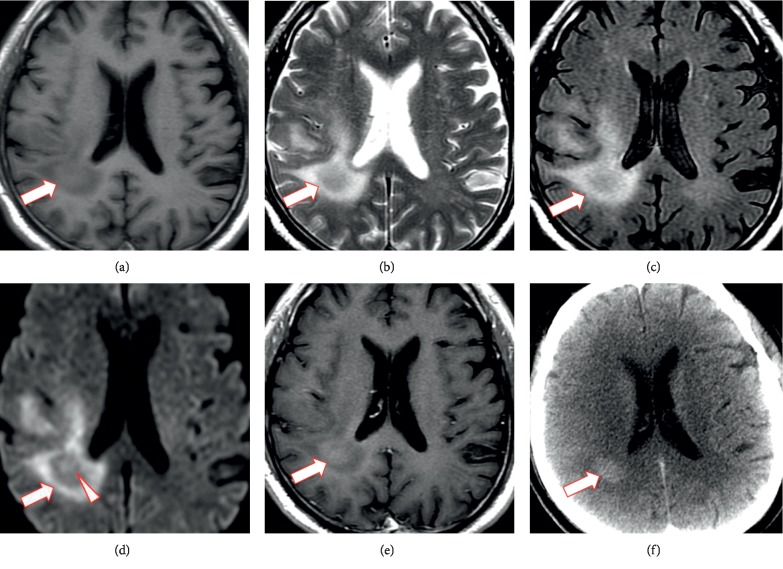
Magnetic resonance and computed tomography images of the brain. (a) Axial T1-weighted, (b) axial T2-weighted, (c) fluid-attenuated inversion recovery (FLAIR), (d) diffusion-weighted, and (e) contrast-enhanced magnetic resonance images display a poorly demarcated lesion in the right temporal and occipital lobe. The lesion shows hypointensity to isointensity on T1-weighted imaging and inhomogeneous hyperintensity on T2-weighted and FLAIR imaging with diffuse perilesional edema. The diffusion-weighted image shows an isointense lesion with a hyperintense region (arrowhead). Postcontrast magnetic resonance imaging shows no enhancement. (f) Axial-view head-window unenhanced computed tomography image of the head shows a hyperattenuated region (arrow).

**Figure 2:**
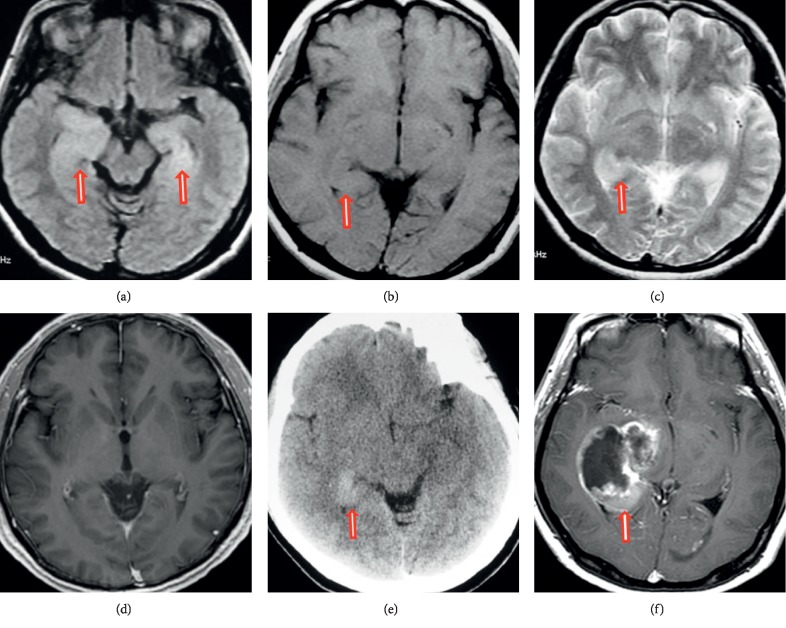
Magnetic resonance and computed tomography images of the brain. (a) Axial fluid-attenuated inversion recovery (FLAIR), (b) T1-weighted, (c) T2-weighted, and (d) contrast-enhanced magnetic resonance images show a poorly demarcated lesion involving the bilateral temporal lobe and hippocampus. The lesion shows homogeneous isointensity on T1-weighted imaging and homogeneous hyperintensity on T2-weighted and FLAIR imaging without perilesional edema or mass edema. The diffusion-weighted image shows an isointense lesion with a hyperintense region. Postcontrast magnetic resonance imaging shows no enhancement. (e) Axial-view head-window unenhanced computed tomography images of the head show a hyperattenuated region (arrow). (f) At the 7-week follow-up, the contrast-enhanced T1-weighted image shows a markedly larger ring and heterogeneously enhanced mass.

**Figure 3:**
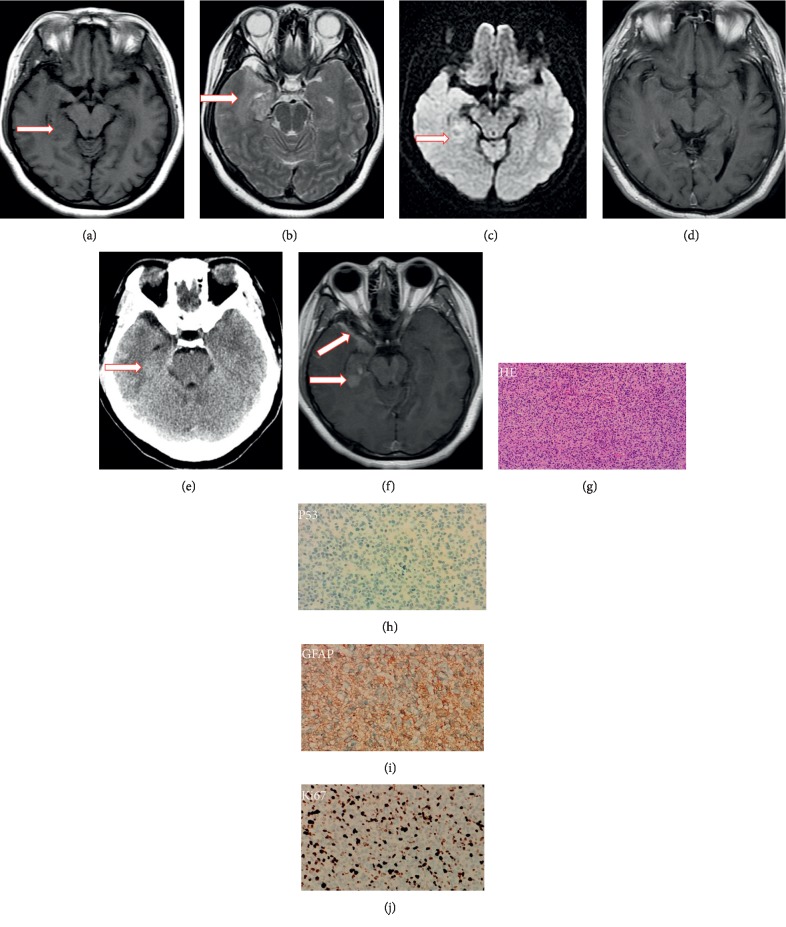
Magnetic resonance and computed tomography images of the brain and histopathological findings of the lesion. (a) T1-weighted, (b) T2-weighted, (c) diffusion-weighted, and (d) contrast-enhanced magnetic resonance images show a poorly demarcated lesion in the right temporal lobe and hippocampus. The lesion shows homogeneous hypointensity on T1-weighted imaging and homogeneous hyperintensity on T2-weighted imaging relative to the brain without perilesional edema or mass edema. The diffusion-weighted image shows an isointense lesion with ring-like peritumoral hyperintensity. Postcontrast magnetic resonance imaging shows no enhancement. (e) Axial-view head-window unenhanced computed tomography images of the head show a hypoattenuated lesion with an isoattenuated region (arrow) in the right temporal lobe. (f) At the 4-week follow-up, a contrast-enhanced T1-weighted image shows a markedly heterogeneous enhanced mass lesion with adjacent pachymeningeal enhancement. (g) Histopathological examination shows proliferation of atypical cells with irregular cytoplasm and chromatin-condensed heterogeneous nuclei, necrotic changes, and microvascular proliferation (hematoxylin and eosin, ×100). (h) A small number of p53-positive cells are present. (i) Glial fibrillary acidic protein immunostaining is positive. (j) Ki-67 positivity is seen in 30% of all cells.

The MR imaging and CT findings for the eight patients in our study are summarized in Table 1. Two lesions were located in the right temporal lobe, two in the left temporal lobe, one the right temporal and occipital lobe, one in the right temporal lobe and hippocampus, one in the bilateral temporal lobe and hippocampus, and one in the brain stem and cerebellum. Three patients had a solitary lesion and five had multiple lesions. Axial T1WI and T2WI and contrast-enhanced images exhibited poorly demarcated lesions (Figures 1–3). The lesions were isointense (Figures 2(b) and 3(a)) (*n* = 2), hypointense (*n* = 5), or of mixed intensity (Figure 1(a)) (*n* = 1) on T1WI and showed homogeneous hyperintensity (Figures 2(a), 2(c) and 3(b)) (*n* = 2) or inhomogeneous hyperintensity (Figures 1(b) and 1(c)) (*n* = 6) on T2WI. Four lesions were associated with perilesional edema (Figures 1(a)–1(d)). Postcontrast MR scans of all lesions displayed no enhancement. DWI (*n* = 7) showed an inhomogeneous hyperintense lesion (*n* = 1) or isointense lesion with a hyperintense region (Figures 1(d) and 3(c)) (*n* = 6). The axial head-window nonenhanced CT images showed a hypoattenuated lesion with a hyperattenuated region (Figures 1(f) and 2(e)) (*n* = 7) or isoattenuated region (*n* = 1). On DWI, the CT hyperattenuated region or isoattenuated region appeared as an inhomogeneous hyperintense lesion (*n* = 1), isointense lesion with hyperintense region (Figure 1(d)) (*n* = 3), or ring-like peritumoral hyperintensity (Figure 3(c)) (*n* = 3). Apparent diffusion coefficient maps showed low-signal intensity in the corresponding hyperintense regions. At the follow-up examination 4 to 15 weeks later, contrast-enhanced T1WI showed a markedly larger ring and heterogeneously enhanced mass.

The final diagnosis of glioblastoma was confirmed by histopathological examination based on the World Health Organization criteria [14]. Histopathologically, the lesions mainly included pseudopalisading necrosis formation and microvascular proliferation in all eight patients. Typical histopathological findings of glioblastoma are shown in Figure 3(g). Five cases were p53-positive and three were p53-negative (Figure 3(h)). Glial fibrillary acidic protein (Figure 3(i)) and S-100 immunostaining were positive in all eight cases. DH1-R132H and 1p19q LOH in fluorescence *in situ* hybridization and incorporation combined with immunostaining were negative in all eight cases. The average Ki-67 labeling index was 29.7% (range, 5%–50%) (Figure 3(j)). These immunohistochemical findings showed that both wild-type IDH1 and normal 1p/19q status supported a diagnosis of *de novo*-type (primary) glioblastoma in all patients [15].

## 4. Discussion

Our retrospective study showed that the MR imaging findings of glioblastoma were isointensity to hypointensity on T1WI, hyperintense ill-defined lesions on T2WI, little or no mass edema, and no contrast enhancement. The CT finding of a hyperattenuated or isoattenuated region combined with the DWI finding of the same region containing an inhomogeneous hyperintense lesion or isointense lesion with a hyperintense region may be specific for a diagnosis of early-stage glioblastoma. This study also suggests that MR imaging has excellent follow-up performance. During follow-up, we observed the development of typical diagnostic characteristics such as a heterogeneously enhanced bulky mass with central necrosis [2, 7].

Patients with a wide range of ages can develop glioblastoma [3, 4, 7]. In this study, the patients' mean age was 55.3 years (range, 36–71 years); additionally, the female : male ratio was 1.7 : 1.0. As described in previous reports [3, 4], most glioblastomas show no specific clinical symptoms. The most common clinical symptoms are seizure and focal signs (e.g., dysesthesia and hemiparesis), and some patient experience vertigo and headache or no symptoms.

According to previous reports [3, 7] and the findings of our study, early-stage glioblastoma is characterized by MR imaging findings of hypointensity of T1WI, hyperintense ill-defined lesions on T2WI, little or no brain parenchyma edema, and no contrast enhancement. A subgroup of glioblastoma is found at an earlier stage, before central necrosis and lesion enhancement occur [3], and about 4% of glioblastoma masses display nonenhancement at the first appearance; these lesions can be misdiagnosed as nonneoplastic cerebral lesions such as infarction, encephalitis, or demyelinating disease [2, 4–7]. Accordingly, two of our patients were considered to have a low-grade glioma based on the initial MR imaging appearance, and the other six patients were considered to have nonneoplastic lesions. Although nonneoplastic lesions (such as multiple sclerosis) may be indistinguishable from early-stage glioblastoma, a solitary ill-defined lesion suggests a brain neoplasm [16, 17]. Most of our patients (5/8) did not have a solitary lesion; however, all had ill-defined lesions, which may be a suggestive sign. Overlap in signal intensity exists between nonneoplastic lesions and glioblastoma [6, 7, 12]. Nonspecific routine MR imaging features can differentiate neoplastic lesions and tumors, and their diagnostic accuracy decreases when used as diagnostic criteria. Therefore, a new diagnostic method is needed to interpret these lesions with an atypical appearance on MR imaging.

The diagnosis of early-stage glioblastoma based on its appearance on routine MR imaging can be challenging. However, early diagnosis and treatment involving gross total excision are likely to prolong the progression-free and overall survival periods [18]. A previous study showed that DWI was useful for diagnosis of malignant glioma [13]. DWI with increased intensity and apparent diffusion coefficient maps showed low signal intensity, which represents low water diffusion and thus suggests high cellularity [18]. Seven of our patients who underwent DWI showed an inhomogeneous hyperintense lesion or isointense lesion with a hyperintense region. A hyperintense area in DWI suggests high cellularity; therefore, this DWI appearance is highly suggestive of a malignant tumor. Although nonneoplastic cerebral lesions such as infarction, encephalitis, and demyelinating or degenerative diseases also frequently show CT hypoattenuation [8–12], these lesions are not associated with regions of hyperattenuation. Glioblastoma is more likely to be inhomogeneous, and unenhanced CT shows a mostly hypoattenuated lesion with hyperattenuated or isoattenuated regions reflecting its histological heterogeneity [6]. Additionally, the CT finding of a hyperattenuated or isoattenuated region combined with a DWI finding of the same region showing an entirely or partly hyperintense lesion supports a tumor diagnosis [12, 13, 18]. The inhomogeneous signal intensity on DWI and inhomogeneous density on CT images may reflect different cell densities and neovascularization [19, 20]. According to past studies of tumor heterogeneity, this feature suggests that high-grade gliomas can appear heterogeneous in terms of their density or signal [20, 21]. Therefore, we consider that a combination of DWI and unenhanced CT findings provides a high level of diagnostic accuracy for early-stage glioblastoma and will help to avoid a delayed diagnosis of glioblastoma and a consequently poor outcome.

Advanced MR imaging approaches might help to differentiate glioblastoma from nonneoplastic cerebral lesions, but they may not offer a complete answer to the diagnostic question of how to differentiate these two conditions. Increased accumulation of *N*-isopropyl-*p*-[123I]-iodoamphetamine on brain single-photon emission tomography [22] is detected in both nonneoplastic cerebral lesions and brain tumors. On MR spectroscopy, nonneoplastic cerebral lesions and tumors may have a similar appearance [23, 24]. Overlap of the advanced imaging appearance of nonneoplastic cerebral lesions and glioblastoma appears to exist. Thus, DWI combined with unenhanced CT may also have a cost-effective and diagnostic role in this condition.

Early-stage glioblastoma may be difficult to differentiate from a nonneoplastic lesion. Close follow-up of these patients is very important. Within several months, these masses progress to typical MR imaging features such as a heterogeneous-enhanced bulky mass with central necrosis, which is more easily diagnosed. Based on previous studies, the average time from the initial scan to final diagnosis of glioblastoma is 4.5 months (range, 1.25–10 months) [3, 25–28]. The duration of clinical symptoms of most patients with primary glioblastoma (68%) is 3 months, and the mean time is 6.3 months [29]. These results are similar to the findings of our study.

## 5. Conclusions

In conclusion, the appearance of early-stage glioblastoma on MR imaging is characterized by a small, ill-defined, isointense to hypointense lesion on T1WI and hyperintense lesion on T2WI, with little or no mass edema and no contrast enhancement. Within several months, this appearance can progress to a bulky mass with enhancement that is typical of glioblastoma. As mentioned above, it is not easy to differentiate early-stage glioblastoma from nonneoplastic lesions using routine MR imaging. However, the combination of a hyperattenuated or isoattenuated region on CT with a region that appears hyperintense in the lesion or part of the lesion on DWI is highly specific for the diagnosis. DWI plus unenhanced CT is more accurate than routine MR imaging alone for diagnosis of early-stage glioblastoma. This is very important because of the extremely poor prognosis, particularly for *de novo* glioblastoma. This diagnostic method offers a survival benefit because of the increased potential for total excision when the mass is still small.

## Figures and Tables

**Table 1:** Imaging findings in eight patients with early-stage glioblastoma.

Patient/age (year)/sex		CT		MR imaging		
	Location	Attenuation	T1-weighted image	T2-weighted image	Diffusion-weighted imaging	Enhancement
1/36/M	Right temporal lobe	A	HYPO	Inhomogeneous HYP	A	No
2/57/F	Right temporal and occipital lobe	A	MIX	Inhomogeneous HYP	B	No
3/41/F	Right temporal lobe and hippocampus	B	ISO	Inhomogeneous HYP	C	No
4/65/M	Bilateral temporal lobe and hippocampus	A	ISO	Homogeneous HYP	No examination	No
5/43/F	Right temporal lobe	A	HYPO	Inhomogeneous HYP	C	No
6/71/F	Brainstem and cerebellum	A	HYPO	Inhomogeneous HYP	B	No
7/62/F	Left temporal lobe	A	HYPO	Inhomogeneous HYP	B	No
8/67/M	Left temporal lobe	A	HYPO	Homogeneous HYP	C	No

CT Attenuation: A, hypoattenuated lesion with hyperattenuated region; B, hypoattenuated lesion with isoattenuated region. Diffusion-weighted imaging: A, hyperintense lesion; B, isointense lesion with hyperintense region; C, isointense lesion with peritumoral hyperintensity. ISO, isointense; HYP, hyperintense; HYPO, hypointense; MIX, mixed isointensity and hypointensity.

## Data Availability

The data used to support the findings of this study are available from the corresponding author upon request.
